# Active Surface with Dynamic Microstructures and Hierarchical Gradient Enabled by in situ Pneumatic Control

**DOI:** 10.3390/mi11110992

**Published:** 2020-11-04

**Authors:** Jian-Nan Wang, Benfeng Bai, Qi-Dai Chen, Hong-Bo Sun

**Affiliations:** 1 State Key Laboratory of Precision Measurement Technology and Instruments, Department of Precision Instrument, Tsinghua University, Haidian District, Beijing 100084, China; jnwang18@mail.tsinghua.edu.cn (J.-N.W.); baibenfeng@tsinghua.edu.cn (B.B.); hbsun@tsinghua.edu.cn (H.-B.S.); 2 State Key Laboratory of Integrated Optoelectronics, College of Electronic Science and Engineering, Jilin University, 2699 Qianjin Street, Changchun 130012, China

**Keywords:** dynamic microstructure, pneumatic, active surface, gradient

## Abstract

An active surface with an on-demand tunable topography holds great potential for various applications, such as reconfigurable metasurfaces, adaptive microlenses, soft robots and four-dimensional (4D) printing. Despite extensive progress, to achieve refined control of microscale surface structures with large-amplitude deformation remains a challenge. Moreover, driven by the demand of constructing a large area of microstructures with increased complexity—for instance, biomimetic functional textures bearing a three-dimensional (3D) gradient—novel strategies are highly desired. Here, we develop an active surface with a dynamic topography and three-tier height gradient via a strain-tunable mismatching-bonding process. Pneumatic actuation allows for rapid, reversible and uniform regulation of surface microstructures at the centimeter scale. The in-situ modulation facilitates large-amplitude deformation with a maximum tuning range of 185 μm. Moreover, the structural gradient can be modulated by programming the strain value of the bonding process. With our strategy, another two types of surfaces with a four-tier gradient and without gradient were also prepared. By providing active modulation and design flexibility of complicated microstructures, the proposed strategy would unlock more opportunities for a wealth of novel utilizations.

## 1. Introduction

The ability to regulate surface topography and adapt to different usage scenarios is of vital importance in various fields including reconfigurable metasurfaces [1,2], adaptive microlenses [3], soft robots [4], wearable devices [5,6], and biomimetic four-dimensional (4D) μ-printing [7,8]. Therefore, active surfaces with dynamic topographies have attracted considerable attention in recent years. Currently, several kinds of strategies have been proposed to regulate surface structure. Examples are surface reconfiguration based on programmable materials (e.g., shape memory polymers (SMPs) [9]), micromorphology adjustment derived from stimuli-responsive materials (SRMs) [10], mechanical tuning using an origami/kirigami technique [11,12], stretching [5,13] and microelectromechanical systems (MEMSs) [14,15]. Despite enormous development, achieving surface reconfiguration with a large variation range, especially for the refined control of microscale structures over a large area, remains a challenge. Moreover, toward practical utilizations, there is an urgent calling for developing manipulation strategies that are easy to operate, user-friendly and respond fast.

On the other hand, for structure engineering, researchers are always fascinated by the sophisticated architectures found in nature, which endow natural materials with unique properties and functions [16,17,18]. For instance, butterflies have evolved overlapping microscales on their wings [17,19], which permits directional adhesive properties to protect the body from being wetted. A recent study reveals that the ultrafast liquid transport on the Sarracenia trichome surface, considered as the source of insect-trapping function, is attributed to the regular arrangement of microribs with two-order hierarchy in height [20]. Nevertheless, to replicate these architecture features in nature is not an easy task. The challenge lies in the search of rational approaches that enables a large-area fabrication of microstructures with complex three-dimensional (3D) distributions, which is hard to realize via conventional planar technologies (e.g., multistep lithography with precise alignment).

Here, we propose a strategy for the facile fabrication and convenient manipulation of an active surface with dynamic microstructures and a tailored hierarchical gradient. A strain-tunable mismatching-bonding process was utilized to simultaneously build an embedded pneumatic network and periodic microstructures with a three-order gradient on the surface. Based on the network, rapid, reversible and uniform regulation of the surface topography was realized at the centimeter scale through pneumatic actuation. The in-situ actuation enables large-amplitude deformation with a maximum tuning range of 185 μm. By combining dynamic modulation and a complicated 3D geometric design, our results would create more opportunities for a variety of utilizations. Moreover, the structural gradient can be readily tailored by regulating the strain of the bonding process. Furthermore, with the proposed strategy, microstructures with well-defined four-tier gradients and without gradient were also successfully obtained.

## 2. Materials and Methods

To design and fabricate a reconfigurable surface, a key point is to rationally establish deformable regions on the surface with a controllable morphing degree. In this work, we prepared a pneumatically actuated surface with an embedded network of microchannels, which served as airways and allowed for tunable deformation under air pumping. By elaborately designing the distribution and sizes of the microchannels, complex surface geometry can be programmed. A strain-tunable mismatch-bonding process was employed to form embedded airtight pneumatic network, and to simultaneously construct centimeter-scale microstructures with a height hierarchy on the surface.

**Figure 1:**
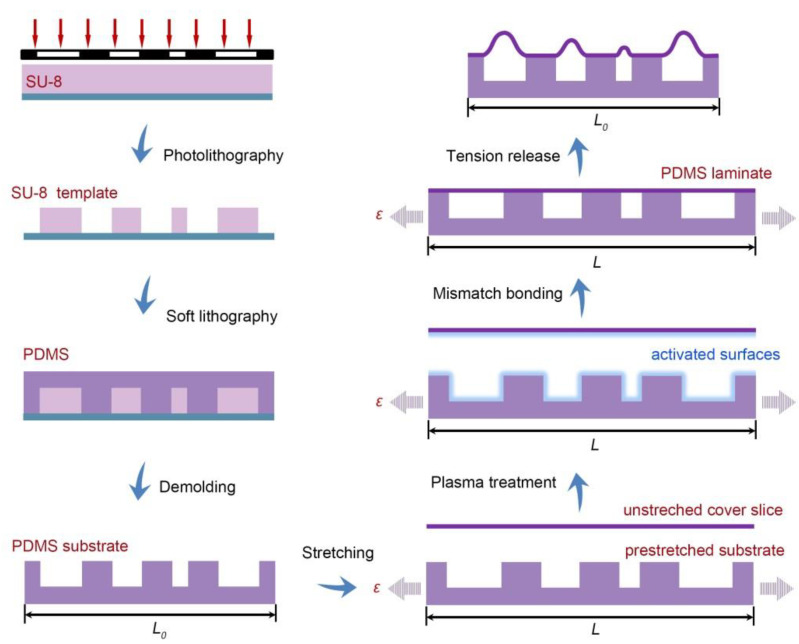
Schematic illustration of the mismatch bonding process for the fabrication of pneumatically actuated surface.

Figure 1 illustrates the fabrication procedure in detail. The pneumatic surface consisted of two elastomer layers, including a structured substrate with microchannels and a cover slice. Both of the two layers were made from polydimethylsiloxane (PDMS) elastomer (Sylgard 184 silicone, Dow Corning, MA, USA) which is widely used in soft-actuated systems and lab-on-a-chip (LoC) devices [21,22,23], due to their high flexibility and ease of processing. The pattern of microchannels was first defined by photolithography. Typically, a layer (150-μm thick) of photoresist (SU-8 2075, MicroChem, MA, USA) was spin-coated on a clean glass slide (pretreated with acetone, ethonal and dionized water), and baked at 95 °C for 2 h. After ultraviolet exposure, the sample was heated at 95 °C for 10 min and developed to obtain microchannel template. Then, the template pattern was transferred to flexible elastomer through soft lithography. PDMS prepolymer was mixed with curing agent (10:1 by weight) and poured into the SU-8 template. After curing at 85 °C for 2 h, the peeled off PDMS layer was used as the elastomer substrate. Simultaneously, the elastomer cover slice was prepared from a spin-coated PDMS thin film (40-μm thick) on a bare glass slide at the same curing condition. To obtain 3D microstructures with height hierarchy, a strain-tunable mismatch-bonding process was adopted to integrate the two layers. The substrate was clamped and prestretched by a home-made stage, while the cover slice remained tension-free. This mismatch in strain was kept in the subsequent plasma treatment and bonding step. The control factor strain (*ε*) is defined by the length variation of the substrate, *ε* = (*L* − *L*_0_)/*L*_0_, where *L*_0_ and *L* are the elastomer lengths before and after stretching (see Figure 1), respectively. By regulating the sample length, tunable strain could be simply applied to the substrate. For reliable manipulation, the applied prestrains in this work were in the range of 35–65%, within the elastic limit. For pneumatic actuation, airtight sealing and a stable interface are essential. Therefore, plasma treatment was implemented to induce strong irreversible bonding between the two layers. Both of them were placed into a plasma cleaner, with the surfaces to be bonded together exposed to the oxygen plasma for 1 min. The modified surfaces were then brought into contact immediately at room temperature for 5 min. Surface oxidation facilitated the formation of covalent siloxane bonds [24,25], which increased the interlayer bonding strength. The cover slice was thus tightly combined to the top regions of the substrate and suspended above the underlying deep microchannels. Owing to the mismatch in strain, release of the tension exerted to the substrate-induced deformation of the cover slice, forming microribs on the surface. By carefully designing the microchannel width, surface geometry with height gradient could be achieved.

The surface morphology of the microstructures was characterized using a JSM-7500 field-emission scanning electron microscope (SEM, JEOL Ltd., Tokyo, Japan). The deformable characteristics under pneumatic actuation were monitored in real time using an optical microscope. To investigate the 3D topography of the dynamic surface in the different states, a LEXT confocal laser scanning microscope (CLSM, OLS4100, Olympus Corp., Tokyo, Japan) was employed.

## 3. Results and Discussion

**Figure 2:**
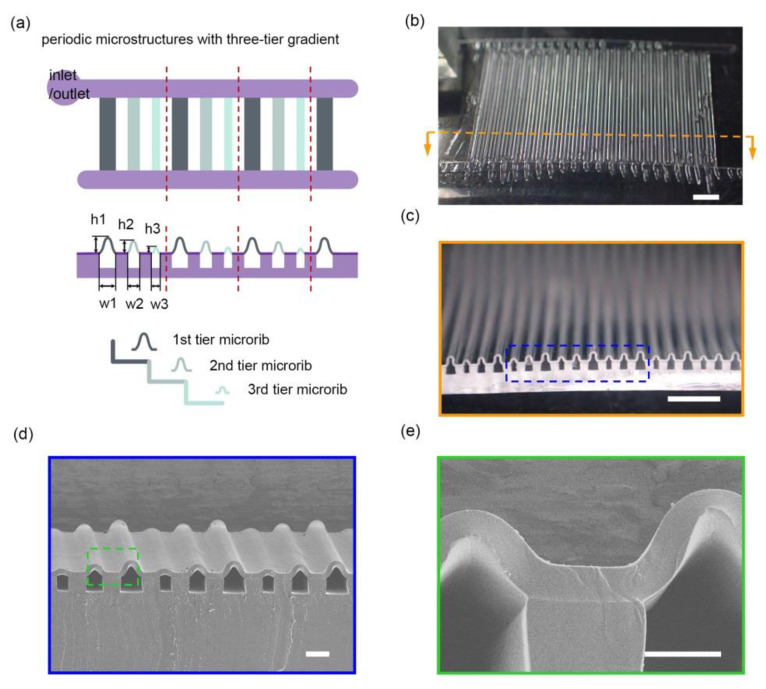
(**a**) Concept of the three-tier gradient. (**b**) Top-view and (**c**) side-view photographs of the fabricated surface. (**d**,**e**) SEM images of (**d**) the periodic gradient microstructures and (**e**) the bonding region. Scale bars: (**b**) 2 mm, (**c**) 1 mm, (**d**) 200 μm, and (**e**) 100 μm.

To illustrate the feasibility of this strategy, periodic microstructures with three-tier gradients were designed and fabricated (see Figure 2). The concept is to convert the horizontal difference in width to the vertical gradient in height, aiming at a facile creation of complicated 3D microstructures. As shown in Figure 2a, three adjacent microchannels which gradually decreased in width (w1 > w2 > w3) is viewed as a group. Taking advantage of the strain mismatch-induced deformation, hierarchy in height (h1 > h2 > h3) is expected to be readily generated on the surface after the bonding process. On the other hand, based on the interconnected pneumatic network, air can be delivered through the whole sample, which makes topography regulation possible. Typically, the widths of the microchannels in this work were set as w1 = 200 μm, w2 = 150 μm, and w3 = 100 μm with an interval of 150 μm under the consideration of operation convenience, mechanical stability and fabrication simplicity. The overall size of the pneumatic surface was 1.8 × 2.2 cm. For practical application, the size can be easily extended to cover larger areas. The hierarchy order and the channel parameters can be also devised at will.

Periodic microstructures were successfully prepared throughout the sample (Figure 2b). The surface morphology exhibited centimeter-scale uniformity, as did the embedded network—shown in the side-view photograph (Figure 2c). As expected, a hierarchical gradient in height was achieved after the bonding process (Figure 2c,d). The SEM image (Figure 2d) further showed that microribs of the same tier were of the same height with similar profile, demonstrating the capability of this strategy to engineer complicated microstructures over a large area. Notably, there was no local collapsing or clogging inside the microchannels (Figure 2c,d), which is important for effective pneumatic control. Another consideration for pneumatic actuation is air leakage, which may lead to operation failure. For this aspect, bonding quality was carefully examined. As the local detail revealed (Figure 2e), the bonding interface was stable without delamination, which effectively avoids air leakage-induced adjacent crosstalk and guarantees a viable pneumatic control.

**Figure 3:**
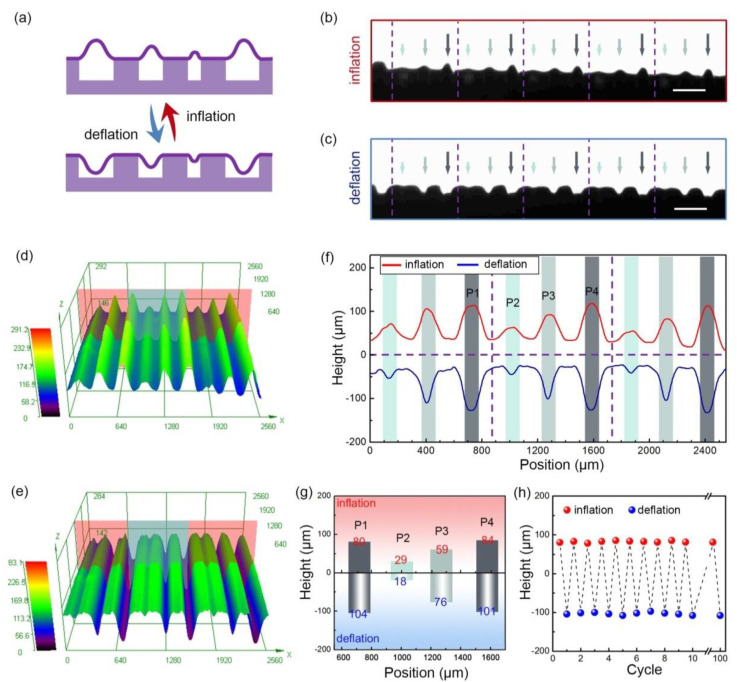
(**a**) Schematic illustration of the dynamic topography regulation. (**b**,**c**) Optical images of the pneumatic surface in the (**b**) inflated and (**c**) deflated states. (**d**,**e**) Three-dimensional (3D) topography of the surface in the (**d**) inflated and (**e**) deflated states. (**f**) Height profiles of the selected zone in (**d**,**e**). (**g**) Height variation of the selected four peaks (P1-P4) in (**f**). (**h**) Pneumatic cycle test. Scale bars: (**b**,**c**) 500 μm.

Based on the pneumatic network with above-mentioned smooth and airtightness, we could dynamically tune the surface topography by pneumatic control (see Figure 3a). The deformation performances in the inflated (Figure 3b) and deflated (Figure 3c) states were monitored in real time using an optical microscope. Five inflation–deflation cycles (10 pneumatic operations) were carried out within 7 s, as shown in Video S1 (the beginning time of each operation was indicated in the video). During each cycle, the surface was fully actuated with a response time less than 1 s. The results indicated that the configuration shifting was fast and reversible within the elastic deformation range, providing a reliable strategy for surface reconfiguration. Moreover, all of the five gradient groups acted in concert without any retardation as shown in Figure 3b,c, illustrating a uniform deformation and large-area controllability of the pneumatic active surface.

Besides fast, reversible and uniform control, a unique feature of this strategy is its capability of modulating the surface microstructures in situ. The dynamic tuning performance at the microscale was carefully investigated using confocal laser scanning microscopy. Figure 3d,e recorded the transient 3D morphologies of the active surface in the inflated and deflated states, respectively. The deformation characteristics of three typical gradient groups indicated the synchronization feature of the regulation process, which further validates the uniform control of microtextures. Moreover, the in-situ modulation feature could be clearly showcased in the comparison to the height profiles in different states (Figure 3f), by monitoring the same deformation zone in Figure 3d,e. The most deformable regions in the inflated state coincide with those in the deflated state for all the hierarchy orders, as denoted by the color bars in Figure 3f. Benefitting from the in-situ reconfiguration, large deformation with a maximum variation range of 185 μm was acquired at position P4 (Figure 3g). The three-tier gradient was also quantitatively characterized by recording the height values of the selected four points (P1-P4) in Figure 3f. Pneumatic regulation enables fine tuning of the surface hierarchical gradient. A larger height difference (83 μm) was obtained in the deflated state, while a difference of 55 μm was found for the inflated state (Figure 3g).

Toward practical applications, a stable performance was highly desired especially for dynamic surface with shape-shifting elements. To further study the stability of this in-situ manipulation, point 4 (P4) on the surface (marked in Figure 3f) was selected as a reference point (any other point is acceptable). Continuous height measurements (Figure 3h) at P4 indicated that the prepared surface could withstand at least 100 pneumatic cycles without apparent signs of fatigue, demonstrating the reliability of this strategy. Another important consideration for reliable control is the selection of cover slice thickness, which gave a direct effect on the surface morphology and dynamic performance (Figure S1). After optimization, 40 μm was selected as the optimum thickness to afford sufficient stiffness and excellent flexibility.

**Figure 4:**
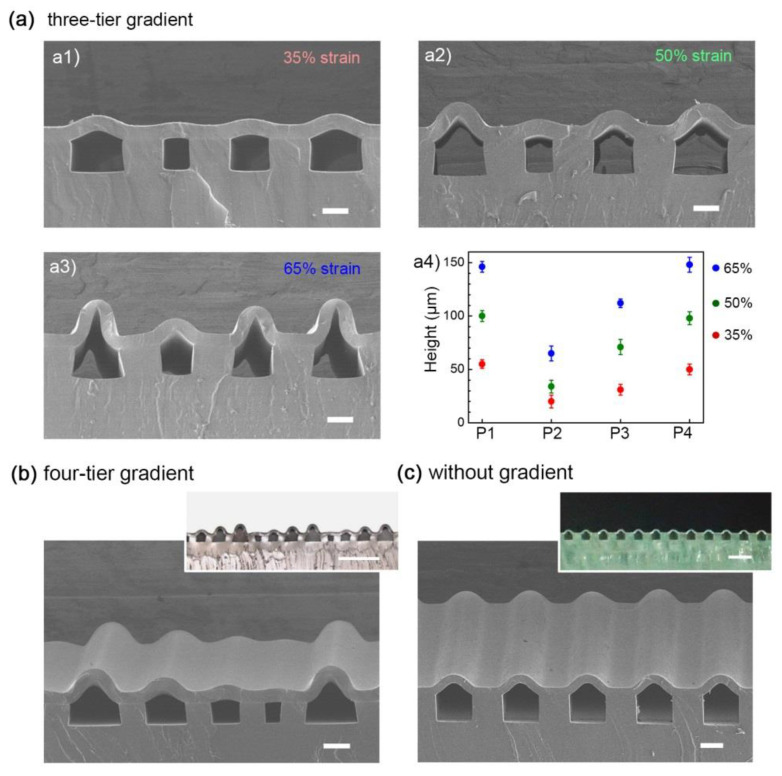
(**a1**–**a3**) Gradient modulation by tuning strains and (**a4**) the corresponding height modification. (**b**,**c**) Another two typical surfaces with (**b**) four-tier gradient and (**c**) without gradient prepared by the method. Scale bars: (**a**–**c**) 100 μm, (inset) 500 μm.

As mentioned above, gradient structures can be readily achieved by a mismatch-bonding process. To further explore the versatility of this strategy, libraries of diverse gradient structures were fabricated by readily tuning the parameters of the bonding process. For instance, strain modulation allowed for refined modification of the three-order height gradient. By using the same substrate template, 3D microstructures with different morphing degrees were produced at 35%, 50% and 65% strain, respectively, as shown in Figure 4(a1–a3). Especially for the high strain value (65%) case, the well-bonded interface without delamination (Figure 4(a3)) demonstrated the potential of the method for fabricating surfaces with a larger deformation amplitude. The heights of the prepared series of gradient microstructures were recorded in Figure 4(a4), exhibiting a significant tuning range acquired from acceptable strain values. Therefore, by controlling the bonding process, various types of gradient can be easily obtained as desired.

In addition to the three-tier gradient microstructures, this strategy can be readily extended to fabricate other types of structured surfaces, such as four-tier gradient microstructures with large-area uniformity (Figure 4b). In this way, by rationally designing the parameters (e.g., width) of the microchannels, microstructures with diverse structural gradient can be flexibly designed and readily fabricated. Notably, the construction of periodic microstructures with uniformity at the centimetre scale validates the reproducibility of this engineering strategy. Apart from different kinds of gradient microstructures, non-gradient microstructures were created as well with the proposed method (Figure 4c).

**Figure 5:**
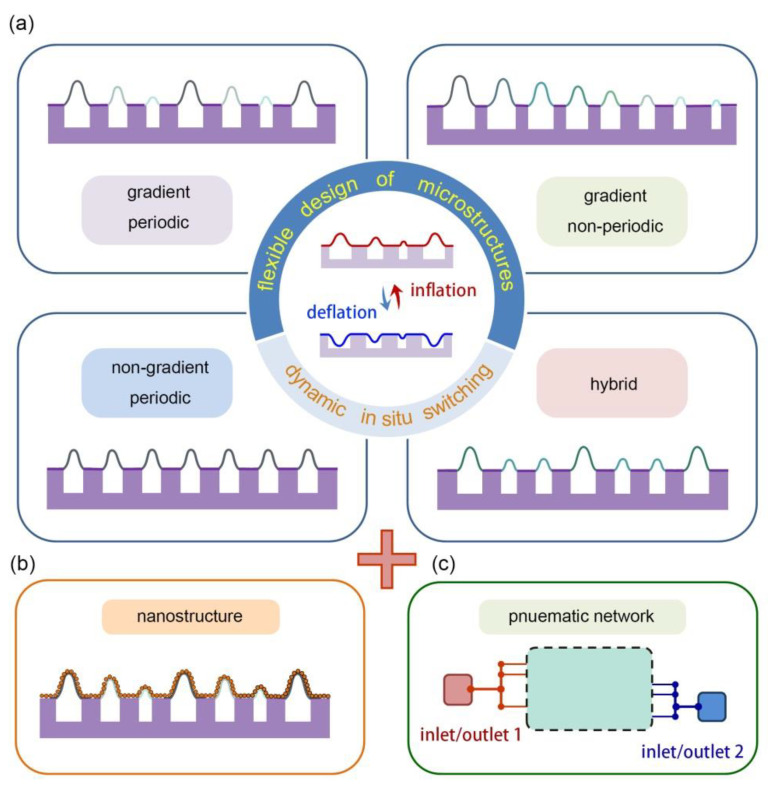
Outlook of the proposed technique from the aspects of (**a**) microstructure design, (**b**) nanostructure modification, and (**c**) pneumatic network arrangement.

For further improvement toward future directions and applications, we give a short outlook of the proposed technique from three aspects. (1) Microstructure design: Considering the realization of the periodic surface, it can be envisaged that a non-periodic gradient surface (Figure 5a) can be also achieved, since it can be viewed as a special case for a periodic surface with only one period. Moreover, the height variation tendency (fast or slow) can be adjusted by tuning the microchannel interval (small or large). The method provides an alternative tool for the large-area fabrication of microstructures with complex 3D distributions, including hierarchical gradient, non-gradient, non-periodic, and the hybrid type combined by the former three types (Figure 5a). (2) Nanostructure modification: The microstructured surface could be further modified with nanostructures (e.g., nanoparticles, nanocoatings, and nanopatterns) [26,27], as presented in Figure 5b. In this way, this strategy would provide a platform for dynamic control at the nanoscale. The integration of the micro- and nanostructures may provide more opportunities for exploring novel utilizations. (3) Pneumatic network distribution: The above two aspects are from the viewpoint of structural design. Further control over the dynamic surface could be achieved by rational design of the airways [28]. For example, the addition of a second inlet/outlet may enable local manipulation of the surface structures, and thus generate an active addressable matrix (Figure 5c). Soft-actuated material with high operation freedom may find multiple uses in the future.

## 4. Conclusions

In conclusion, an active surface with reconfigurable microstructures and a hierarchical gradient has been readily prepared, flexibly programmed, and in-situ manipulated for large-amplitude control. A mismatch-bonding process was employed to construct centimeter-scale surface microstructures with a three-tier gradient in a facile way, and to simultaneously form an embedded airtight network of interconnected microchannels which greatly contributes to excellent large-area deformability. Based on the network, rapid, reversible, and uniform regulation of surface topography has been demonstrated under pneumatic actuation. Taking advantage of the in-situ actuation mode, large deformation with a maximum amplitude of 185 μm was achieved. Moreover, the strain-tunable bonding process allows for the fine tuning of the gradient parameters using one substrate template. To further explore the design flexibility of the proposed strategy, we also prepared microstructured surfaces with four-tier gradients and without gradients as two typical examples. By providing liable active manipulation and a wide design space of intricate microstructures, our results would unlock more options for the creation of multifunctional reconfigurable surfaces and exploration of novel applications. As proved by recent studies, stable topology regulation is of great value for adaptive materials [29], reconfigurable metasurfaces [1,30], shape-morphing materials [31], active control of biofouling [32], and so on. Microstructures with hierarchical organization can trigger unique high-speed liquid transportation [20]. Accordingly, the huge potential of the proposed strategy would be showcased in the future.

## Supplementary Materials

The following are available online at https://www.mdpi.com/2072-666X/11/11/992/s1, Video S1: Surface topography reconfiguration under pneumatic actuation. Figure S1: Microstructures fabricated using PDMS slices with different thickness.
