# Supplementary material for: Active Surface with Dynamic Microstructures and Hierarchical Gradient Enabled by in situ Pneumatic Control
Source: Micromachines (Basel). 2020 Nov 4;11(11):992. doi: 10.3390/mi11110992 (PMC7694221; doi:10.3390/mi11110992)
Supplement: Supplementary file 1 [file micromachines-11-00992-s001.zip › supplementary/Supplementary Material.pdf]

Supplementary Material

Active Surface with Dynamic Microstructures and Hierarchical Gradient Enabled by in situ Pneumatic Control

Jian-Nan Wang [1,2], Benfeng Bai [1], Qi-Dai Chen [2,*] and Hong-Bo Sun [1,2]

[1] State Key Laboratory of Precision Measurement Technology and Instruments, Department of Precision Instrument, Tsinghua University, Haidian District, Beijing 100084, China; jnwang18@mail.tsinghua.edu.cn (J.-N.W.); baibenfeng@tsinghua.edu.cn (B.B.); hbsun@tsinghua.edu.cn (H.-B.S.)

[2] State Key Laboratory of Integrated Optoelectronics, College of Electronic Science and Engineering, Jilin University, 2699 Qianjin Street, Changchun 130012; chenqd@jlu.edu.cn (Q.-D.C.)

* Correspondence: chenqd@jlu.edu.cn (Q.-D.C.)

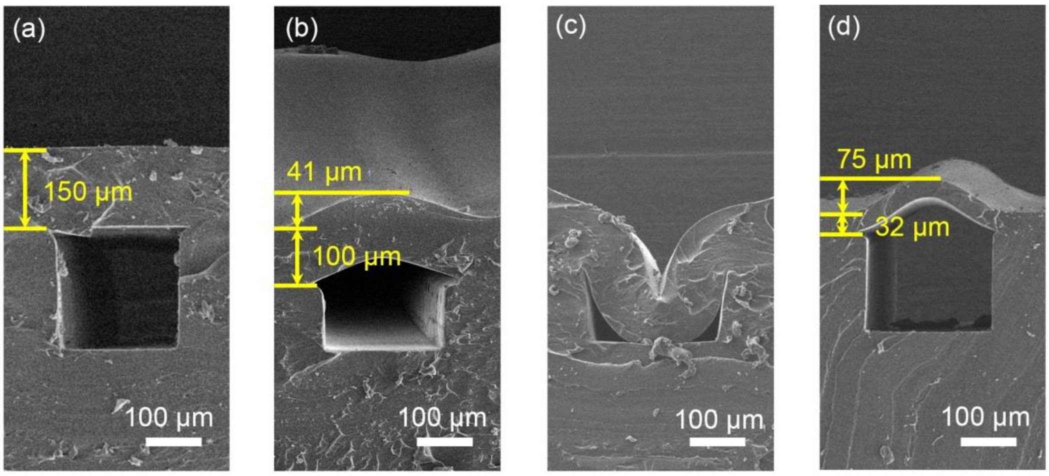

Figure S1. Microstructures fabricated using PDMS slices with different thickness. (a) 150 µm, (b,c) 100 µm, (d) 32 µm. All of the samples were prepared under 40% strain. The widths of microchannels in (a-d) were 200 µm.

Note

The cover slice served as a deformable layer, therefore, the thickness selection of

PDMS slice gave a direct effect on the surface morphology and dynamic performance. Under the same strain value (40%), a series of microstructures were prepared using PDMS slices of different thickness including 150 μm, 100 μm, and 32 μm. The heights of the obtained microstructures (microchannel width, 200 μm) were 4 μm (Figure S1a), 41 μm (Figure S1b), and 75 μm (Figure S1d), respectively. As shown in Figure S1a, the use of thick sheet leaded to the formation of a nearly flat surface due to the dramatic deformation resistance. When the slice thickness decreased to 100 μm (Figure S1b), the prepared surface was locked in the microchannel after deflation (Figure S1c) and lost the response characteristic thereafter. While for the sample prepared with thin PDMS (Figure S1d), despite of the excellent deformation capability, it was too tender to withstand frequent operations. Based on the above results, 40 μm was selected as the optimum thickness to afford sufficient stiffness and excellent flexibility.
